# Heavy and moderate interval exercise training alters low-flow-mediated constriction but does not increase circulating progenitor cells in healthy humans

**DOI:** 10.1113/expphysiol.2011.062836

**Published:** 2011-12-16

**Authors:** Mark Rakobowchuk, Emma Harris, Annabelle Taylor, Vivek Baliga, Richard M Cubbon, Harry B Rossiter, Karen M Birch

**Affiliations:** Multidisciplinary Cardiovascular Research Centre, University of LeedsLeeds, UK

## Abstract

Moderate-intensity endurance exercise training improves vascular endothelial vasomotor function; however, the impact of high-intensity exercise training has been equivocal. Thus, the effect of the physiological stress of the exercise remains poorly understood. Furthermore, enhanced vascular repair mediated by circulating progenitor cells may also be improved. To address whether the physiological stress of exercise training is an important factor contributing to these adaptations, 20 healthy participants trained for 6 weeks. Training involved either moderate (MSIT; *n*= 9) or heavy metabolic stress (HSIT; *n*= 11) interval exercise training programmes matched for total work and duration of exercise. Before and after training, flow-mediated dilatation, low-flow-mediated constriction and total vessel reactivity were measured at the brachial artery using Doppler ultrasound. Circulating progenitor cells (CD34^+^, CD133^+^ and CD309/KDR^+^) were measured by flow cytometry (means ± SD). Relative (MSIT pre- 5.5 ± 3.4 *versus* post-training 6.6 ± 2.5%; HSIT pre- 6.6 ± 4.1 *versus* post-training 7.0 ± 3.4%, *P*= 0.33) and normalized (*P*= 0.16) flow-mediated dilatation did not increase with either training programme. However, low-flow-mediated constriction was greater after training in both groups (MSIT pre- −0.5 ± 3.2 *versus* post-training −1.9 ± 3.1%; HSIT pre- −1.0 ± 1.7 *versus* post-training −2.9 ± 3.0%, *P*= 0.04) and contributed to greater total vessel reactivity (MSIT pre- 7.4 ± 3.3 *versus* post-training 10.1 ± 3.7%; HSIT pre- 10.9 ± 5.9 *versus* post-training 12.7 ± 6.2%, *P*= 0.01). Peak reactive hyperaemia and the area under the shear rate curve were not different between groups, either before or after training. Although circulating progenitor cell numbers increased following heavy-intensity interval exercise training, variability was great amongst participants [MSIT pre- 16 ± 18 *versus* post-training 14 ± 12 cells (ml whole blood)^−1^; HSIT pre- 8 ± 6 *versus* post-training 19 ± 23 cells (ml whole blood)^−1^, *P*= 0.50]. Overall, vasoconstrictor function may be augmented by moderate- and heavy-intensity interval exercise training in young adults. However, circulating progenitor cell numbers were not increased, suggesting that these cells are not likely to be upregulated as a result of training.

The progression of cardiovascular disease is blunted by regular exercise training, especially endurance training, while physical inactivity is a primary modifiable risk factor (Celermajer *et al.* 1994; Anderson *et al.* 1995; Suwaidi *et al.* 2000). Endothelial dysfunction is known to precede cardiovascular disease; thus, its primary prevention or amelioration through exercise prescription and adherence, when effective and appropriate, may reduce the incidence of cardiovascular disease (Higashi *et al.* 1999; Hambrecht *et al.* 2003; Green *et al.* 2004). High-intensity interval exercise training has been proposed as a ‘time-efficient’ strategy to improve endothelial vasomotor function (Wisloff *et al.* 2007; Rakobowchuk *et al.* 2008), and several studies suggest that it is a more effective training method than traditional moderate-intensity continuous exercise regimes (Gibala, 2007; Wisloff *et al.* 2007; MacDonald & Currie, 2009). Generally, aerobic fitness has been improved in as few as 2 weeks of sprint interval training (Burgomaster *et al.* 2005); however, endothelial improvements are often noted by 4 weeks of training (Tinken *et al.* 2008) and remodelling and normalization of endothelial function may occur beyond 8 weeks (Tinken *et al.* 2008). On the contrary, some studies indicate that high-intensity training may impair endothelial function (Bergholm *et al.* 1999) or at least have no beneficial impact (Goto *et al.* 2003).

Exercise intensity is a relatively poorly defined term, especially when characterized as a function of maximal oxygen uptake (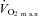). More appropriately, exercise domains related to lactate threshold (LT), critical power (CP) and 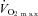 provide a better characterization of the induced physiological stress (Rossiter, 2011). Power output and exercise intensity can be independently modulated by interval duration (Turner *et al.* 2006), with short-duration intervals displaying a lower metabolic stress than longer duration intervals despite the power output, training duration and total work being similar between the two (Turner *et al.* 2006). In the present study, therefore, we aimed to determine the effect of moderate- and heavy-intensity interval exercise training programmes on the parameters of vascular endothelial function and the circulating progenitor cells (CPCs) that are related to vascular repair, angiogenesis and vasculogenesis.

Flow-mediated dilatation (FMD) is a commonly used parameter to evaluate vascular endothelial vasomotor function (Corretti *et al.* 2002; Harris *et al.* 2010; Thijssen *et al.* 2011*a*) and provides prognostic information regarding vascular ‘health’ (Celermajer *et al.* 1994). Recently, evaluation of the endothelium-dependent responses to acute reductions of shear stress (low-flow-mediated constriction; L-FMC) have been made, and this provides an index of the endothelial contribution to resting vasomotor tone (Gori *et al.* 2008). The magnitude of L-FMC is partly determined by the release of endothelin-1 (Spieker *et al.* 2003) or inhibition of the release of endothelium-derived hyperpolarizing factor and cyclo-oxygenase products (e.g. prostaglandins) and may be blunted in disease (Gori *et al.* 2008). Low-flow-mediated constriction is not nitric oxide dependent, which supports the notion that this measure provides a unique assessment of vasomotor function and endothelial health (Gori *et al.* 2010). In addition, L-FMC is not correlated with FMD and shows a clear dose–response relationship with the severity of coronary artery disease in patients (Gori *et al.* 2011). Although endothelial function is improved in many populations with exercise training (Hambrecht *et al.* 1998, 2003; Clarkson *et al.* 1999; Rakobowchuk *et al.* 2008), L-FMC has not been evaluated following exercise training. In addition, the composite measure of total vessel reactivity (combined L-FMC and FMD) has not been assessed following exercise training and may provide useful additional information regarding the prediction of cardiovascular disease risk (Gori *et al.* 2011), although it should be stated that isolated reporting of this composite end-point would mask mechanistic information provided by individual assessment of L-FMC and FMD.

Circulating progenitor cells are thought to be cells of haematopoietic origin (Vasa *et al.* 2001) and may play a paracrine role in vascular proliferation (angiogenesis/vasculogenesis) and possibly vascular endothelial repair (Crosby *et al.* 2000). After an acute exercise bout, these cells are mobilized by a nitric-oxide-dependent mechanism (Cubbon *et al.* 2010) and probably require exercise that induces a high metabolic stress (Adams *et al.* 2004; Sandri *et al.* 2005). Some studies, however, also show acute mobilization of these cells following exercise below LT (moderate exercise domain; Laufs *et al.* 2005; Cubbon *et al.* 2010). From the few training studies that have investigated upregulation of CPCs in healthy populations, results have been contrasting, with some studies suggesting an elevation in CPC numbers (Laufs *et al.* 2005), while others find no change (Thijssen *et al.* 2006). Nevertheless, CPC mobilization following interval training has yet to be determined.

We aimed to determine whether 6 weeks of interval exercise training increased the vascular endothelial function, as determined by elevations in shear-induced vasodilatation and reductions in shear-induced vasoconstriction, and the circulating progenitor cell numbers as a marker of endothelial repair mechanisms. In addition, we examined the influence of differing magnitudes of exercise metabolic stress during training bouts matched for power output, duration and work upon these variables. We hypothesized that following heavy metabolic stress interval training (HSIT), CPC mobilization would be increased to a greater extent than in a moderate metabolic stress interval training (MSIT) group, whereas vascular function would be enhanced (increased FMD and augmented vasoconstriction) to a similar degree as a result of similar overall flow responses.

## Methods

### Participants

Healthy men (*n*= 7) and women (*n*= 13) volunteered for the study (Table 1). The following inclusion criteria were used for the participants: (a) free from apparent cardiovascular, pulmonary or metabolic disease; (b) deemed safe to begin a physical activity programme; and (c) not engaged in a regular exercise training programme as assessed by preparticipation questionnaires and a physical activity readiness questionnaire. In addition, a cardiologist attended the initial aerobic fitness testing and applied the American Heart Association guidelines to begin an exercise programme (Balady *et al.* 1998). Other exclusion criteria included medication use, pregnancy and smoking, as assessed through baseline screening. The experimental procedures and potential risks were explained prior to the study when the participants attended an initial familiarization session, and all participants provided written, informed consent to participate. The Faculty of Biological Sciences Ethics Board at the University of Leeds approved the experimental protocol, which conformed to the Declaration of Helsinki.

**Table 1:** Subject characteristics at rest over the course of 6 weeks of either moderate- or heavy-intensity interval training

	Moderate (*n*= 9)		Heavy (*n*= 11)	
		
	Before	After	Before	After
Male/female	3/6	—	4/7	—
Age (years)	23.7 ± 3.4	—	23.1 ± 2.5	—
Height (cm)	173.9 ± 5.5	—	171.7 ± 12.8	—
Weight (kg)	74.3 ± 12.2	73.2 ± 9.9	67.3 ± 13.6*	67.0 ± 13.3*
Body mass index (kg m^−2^)	24.5 ± 2.2	24.4 ± 3.5	22.8 ± 3.1*	22.8 ± 3.0*
Resting heart rate (beats min^-1^)	71 ± 10	66 ± 11	65 ± 9	64 ± 10
Absolute 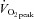 (l min^−1^)	2.58 ± 0.70	2.61 ± 0.56	2.39 ± 0.71*	2.72 ± 0.63†
Relative 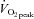 (ml kg^−1^ min^−1^)	34.7 ± 6.5	35.7 ± 5.9	35.7 ± 8.3	41.2 ± 8.1†
Brachial artery baseline diameter (mm)	3.73 ± 0.68	3.69 ± 0.65	3.32 ± 0.67*	3.35 ± 0.65*
Brachial artery maximal diameter (mm)	3.92 ± 0.63*	3.92 ± 0.64*	3.53 ± 0.68	3.59 ± 0.77
Absolute FMD (mm)	0.19 ± 0.10	0.24 ± 0.07	0.21 ± 0.13	0.24 ± 0.11

Data are means ± SD. Abbreviations: FMD, flow-mediated dilatation; and 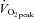, peak oxygen uptake.

* Significant group difference at the same time point (main effect for group, *P* < 0.05).

† Significant difference with training (group × time interaction, *P* < 0.05).

### Experimental protocol

Participants visited the laboratory for baseline assessments prior to completing a 6 week exercise training programme. Initially, resting ECG and vascular measures, and peak oxygen uptake (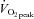) were determined. Participants were then assigned to either a heavy metabolic stress interval training or a moderate metabolic stress interval training group in a matched fashion, based on initial 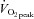. All post-training assessments were repeated between 48 and 72 h after the last training session. Prior to the baseline and post-training measures, participants refrained from ingesting food and caffeine between 8 and 12 h before the test and had not consumed alcohol or participated in exercise within the previous 24 h. Female participants were tested ∼2 weeks prior to the commencement of the training programme to ensure that post-training assessments were within the same phase of their individual menstrual cycle.

### Assessment of vascular function

After 20 min of supine rest in a temperature-controlled room (22–24°C), with the right arm at 90 deg abduction, the participant was instrumented with an ECG (V5 configuration). The same researcher imaged the participant's brachial artery using a 7 MHz linear array ultrasound probe (Aspen, Acuson; Siemens Medical, Camberley, UK) and obtained 20 sequential ECG-gated end-diastolic images using commercially available frame-grabbing software (Vascular Imager; Medical Imaging Applications, Coralville, IA, USA). According to established guidelines (Corretti *et al.* 2002; Thijssen *et al.* 2011*a*), a 5 min forearm ischaemic period, with the cuff placed distal to the ultrasound probe placement, was followed by collection of 180 ECG-gated images. Blood velocity measurements were acquired at 40 MHz throughout the reactive hyperaemia period using a data acquisition system (Powerlab model ML; ADInstruments, Colorado Springs, CO, USA) and software (LabChart 7.2; ADInstruments). Brachial artery diameter was analysed offline using Brachial Tools v.5 (Medical Imaging Applications). Peak diameter was determined from the highest average of three consecutive heart cycles, while baseline diameter was calculated as an average from the 20 baseline images. Continuous blood velocity was determined using a fast Fourier transform weighted for signal intensity and adjusted for insonation angle. Relative FMD, L-FMC and total vessel reactivity (TVR) were calculated as follows:


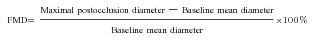



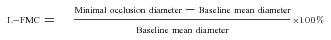



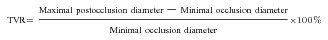


The velocity–time integral (VTI) was determined both for 60 (VTI_60_) and 90 s (VTI_90_) after cuff release, and the area under the shear rate curve (AUC) was calculated as (8 × VTI)/mean brachial baseline diameter for each period. The FMD was normalized using area under the shear rate curve. Peak, 60 s mean and 90 s mean blood velocity and shear rates were also calculated from the velocity profiles. Peak reactive hyperaemia was determined as the maximal velocity after cuff release.

### Enumeration of circulating progenitor cells

Circulating progenitor cells were enumerated using a commercially available kit (EPC enumeration kit; Miltenyi Biotec, Bergish Gladblach, Germany). Briefly, after an overnight fast a 22 ml blood sample from the antecubital vein was collected into EDTA tubes. This procedure was performed following the resting cardiovascular measurements, but prior to the cardiopulmonary exercise assessment. The whole blood was separated into two 10 ml samples and one 0.2 ml sample. All samples were red cell lysed and total leucocytes enumerated by haemocytometry. All samples were centrifuged at 300 *g* for 10 min to form a pellet, and the supernatant was removed. One 10 ml sample was then resuspended in fluorescence-activated cell sorting buffer (FACS buffer: 2 mm EDTA and 1% bovine serum albumin) and incubated with antibodies specific for CD34^+^, CD14^+^, CD309/KDR^+^ and CD133^+^, while the other 10 ml sample pellet was resuspended and incubated with antibodies specific for CD34^+^, CD14^+^, CD133^+^ and an isotype control for CD309/KDR^+^. The CD34^+^ antibodies in these samples were coated with magnetic beads. The 0.2 ml sample was incubated with CD14^+^ and CD34^+^ antibodies and isotype controls for CD133^+^.

The two 10 ml samples were subsequently washed with FACS buffer, centrifuged for 10 min at 300 *g* and resuspended in FACS buffer. MACS magnetic cell separation columns (Miltenyi Biotec, Bergisch Gladbach, Germany) were prepared by washing with FACS buffer, after which the two 10 ml samples were passed through to further isolate CD34^+^ cells. Propidium iodide was added to differentiate dead cells, and the sample was then immediately enumerated by flow cytometry using a FACSCalibur cytometer and CellQuest software (Becton Dickinson, Oxford, UK) with the gating strategy recommended by the manufacturer (Miltenyi Biotec). This method differs from previous work (Vasa *et al.* 2001; Adams *et al.* 2004; Tinken *et al.* 2008) and has recently been shown to follow the time course of CPC mobilization following acute coronary syndrome. The magnetic separation of CD34^+^ cells attempts to isolate specifically the circulating progenitor cells, in comparison to standard methods, which have traditionally enumerated leucocytes expressing up to three endothelial and/or haematopoietic cell surface markers. In addition, the present method uses a more specific gating strategy that takes into account the fact that progenitor cells exhibit low forward and side-scatter and thus appear in the lymphocyte region of a standard flow cytometric dot plot. This method also involves the exclusion of dead cells and CD14^+^ monocytes. Moreover, absolute standardization of all reagents and processing steps, along with the production of an absolute CPC count per millilitre, enables interexperimental comparisons that cannot be offered by the majority of published data.

### Cardiopulmonary exercise assessment

Participants performed a ramp-incremental exercise test (increasing by 1 W every 4 s for women or 1 W every 3 s for men) on an electronically braked cycle ergometer (Excalibur Sport V2.0; Lode BV, Groningen, The Netherlands) to bring subjects to the limit of tolerance within ∼10–15 min. Gas exchange and ventilatory variables were measured breath by breath following calibration according to the manufacturer's instructions (Medgraphics D-Series; Medgraphics, Medical Graphics Corporation, St Paul, MN, USA). The test began with 2 min of seated rest, followed by 2 min of baseline cycling at 20 (women) or 40 W (men). The test was terminated when the participant could no longer sustain a cadence of 50 r.p.m. despite strong verbal encouragement, at which point the work rate was reduced to 20 W to allow 5 min of active recovery. The 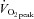 was calculated as the average value over the final 30 s of the ramp-incremental phase. The maximal work rate attained at 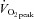 was used to determine the exercise training work rates.

### Exercise training protocol

Participants completed 6 weeks of training, individually attending three supervised sessions per week in the laboratory. Outside the laboratory, participants were asked to refrain from supplemental training or nutritional strategies. The MSIT and HSIT training programmes were based on previous work (Turner *et al.* 2006). Participants completed 30 (weeks 1–2), 35 (weeks 3–4) and 40 min (weeks 5–6) of exercise at each training bout, preceded by a 2 min warm-up at 20 W. The MSIT group completed repeated intervals (10 s:20 s) consisting of 10 s at 120% of their pretraining peak work rate (in the ramp-incremental test) and 20 s of recovery at 20 W. This pattern repeated until the target duration was met. The HSIT group completed intervals of 30 s:60 s in the same manner, and both groups completed their sessions on the aforementioned Lode Excalibur electronically braked ergometer. The protocols involved an identical total training volume, power output and duration, but differed in perceived exertion and metabolic stress (to view example lactate responses to each exercise session, see Turner *et al.* 2006). According to previous work, the MSIT intervals were chosen to maintain a metabolic rate below LT (no appreciable accumulation in blood lactate, similar to constant work rate exercise < LT), while the HSIT intervals would induce a raised, but steady-state, blood lactate response (Turner *et al.* 2006). All participants attended their designated 18 training sessions. It should be noted that an additional eight individuals completed the initial screening and testing sessions, but did not begin training.

### Statistical analysis

Data were assessed for normal distribution using the Kolmogorov–Smirnov test. Baseline data were examined for group differences via Student's unpaired *t* test. Data were then analysed to assess the impact of training using two-way mixed model ANOVA with ‘group’ (HSIT *versus* MSIT) and ‘time’ factors (pre- *versus* post-training). When a significant interaction was noted, Bonferroni-corrected *t* tests were used to determine differences. Pearson correlations were performed on selected variables of interest. Significance for all analysis was accepted as *P*≤ 0.05. All values are presented as means ± SD. Analyses were performed using statistical software (SPSS version 18.0; IBM Corporation, Somers, NY, USA). The CPC data were obtained and analysed from 16 participants (samples from two participants in each group were not analysed owing to unavailability of the flow cytometer). The coefficient of variation for repeated measures of CPCs was 17% (*n*= 6). The day-to-day repeatability (coefficient of variation) for FMD, L-FMC and total vessel reactivity was 15, 20 and 12% (*n*= 8), respectively. The absolute day-to-day difference was 1.1, 1.4 and 1.7%, respectively. The coefficient of variation was calculated as the standard deviation of the measures divided by the mean of the measures × 100%.

## Results

### Participants

Participant details are shown in Table 1, and all participants completed the training programme as outlined. Although participants were matched according to 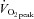, there were some baseline differences in body mass and body mass index, but all values fell within the healthy range for this age group. Both absolute and relative 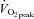 increased with training in the HSIT group but were unaltered in the MSIT group (group × time interaction; absolute 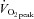, *P*= 0.04; relative 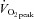, *P*= 0.05; Table 1).

### Velocity–time integral, shear rates following reactive hyperaemia and basal brachial blood flow

**Fig. 1:**
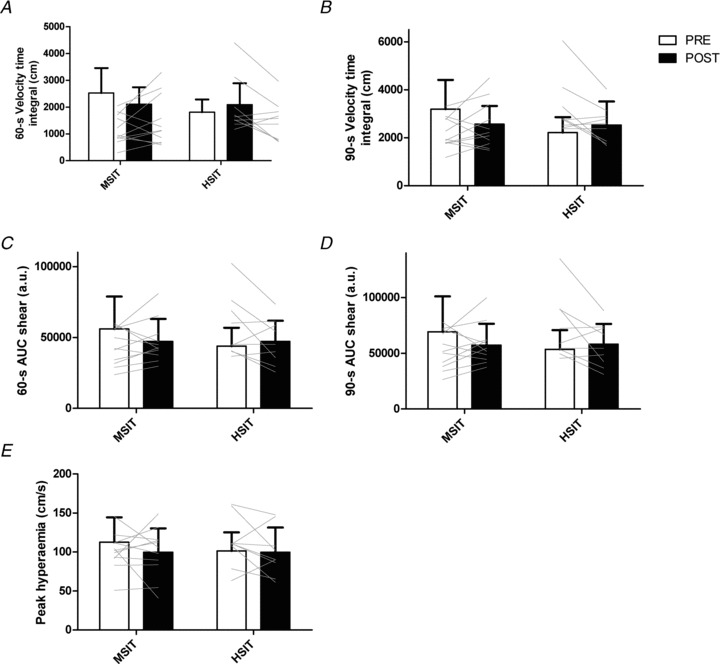
Velocity–time integral (VTI), shear rates and blood flow *A* and *B*, the 60 s velocity–time integral for both the 60 and 90 s periods after cuff release were not significantly changed with training in either group (VTI_60_*P*= 0.63, VTI_90_*P*= 0.42). *C* and *D*, the shear AUC over 60 and 90 s were likewise not significantly changed with either training group (60 s AUC shear *P*= 0.41, 90 s AUC shear *P*= 0.44). Peak hyperaemic blood velocity was also unaltered with either training group (*P*= 0.31).

The VTIs for both the 60 and 90 s periods after cuff deflation were not different between testing sessions, and there was no group × time interaction (time effect, VTI_60_*P*= 0.63, VTI_90_*P*= 0.42; Fig. 1*A* and *B*). Likewise, the shear AUC values over 60 and 90 s were also not changed with training (time effect, 60 s AUC shear *P*= 0.41, 90 s AUC shear_90_*P*= 0.44; Fig. 1*C* and *D*), and no interaction was found. Peak hyperaemic blood velocity was also unaltered with training in both groups (group × time effect *P*= 0.31; Fig. 1*E*). Finally, basal limb blood flow of the brachial artery was elevated with training (HSIT, pretraining 40.5 ± 22.9 and post-training 49.7 ± 29.2 ml min^−1^; MSIT, pretraining 36.0 ± 27.2 and post-training 53.6 ± 34.7 ml min^−1^, time effect *P*= 0.05, no group × time effect *P*= 0.53). There was also a modest correlation between basal brachial blood flow and L-FMC (*r*= 0.42, *P* < 0.01). With training, the difference between basal limb shear and the shear during occlusion increased, but not significantly (pretraining 4.8 ± 13.3 and post-training 9.8 ± 11.5 s^−1^, time effect *P*= 0.16, no group × time effect *P*= 0.70).

### Vascular function assessed by FMD, L-FMC and total vascular reactivity

**Fig. 2:**
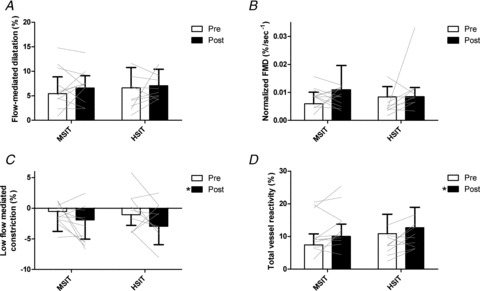
Vascular endothelial function Flow-mediated dilatation (FMD) was not significantly increased with training in either group (*A*; *P*= 0.33), although a trend was observed for normalized FMD (*B*; *P*= 0.16). Conversely, low-flow-mediated constriction (*C*; *P*= 0.048) and total vessel reactivity (*D*; *P* < 0.01) increased with time, but there was no group × time interaction. * Significant main effect for time (*P* < 0.05).

Absolute (Table 1) and relative brachial FMD were unaltered by training in both groups (time effect *P*= 0.33; Fig. 2*A*). A general trend for increased normalized FMD was apparent, but did not reach statistical significance (time effect *P*= 0.16; Fig. 2*B*). The L-FMC increased in a similar fashion with training in both groups (time effect *P*= 0.048, no group × time interaction *P*= 0.78; Fig. 2*C*). The combination of a small increase of FMD and an increase of L-FMC augmented total vessel reactivity with training (time effect *P*= 0.01; Fig. 2*D*), and no interaction was observed (*P*= 0.61).

### Circulating progenitor cell numbers

**Fig. 3:**
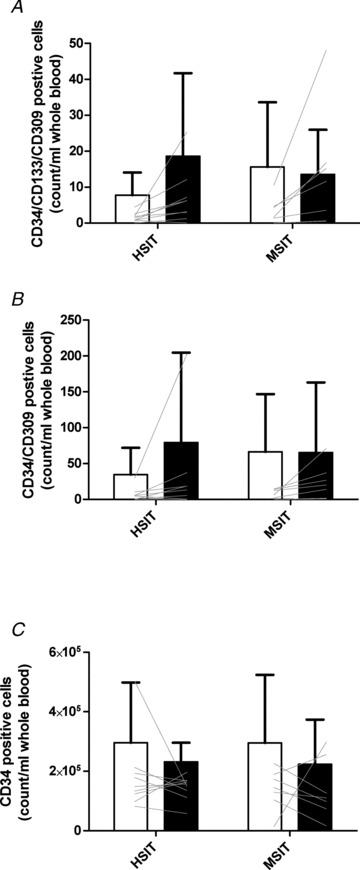
Circulating progenitor cells Individual and mean values of CD34^+^, CD133^+^, CD309^+^ cells (*A*), CD34^+^, CD309^+^ cells (*B*) and CD34^+^ cells (*C*). Note that there was no increase with training and only two participants displayed increases following training.

Different populations of circulating progenitor cells were enumerated, but there were no alterations in these populations in either training group. Numbers of CD34^+^ cells were not altered in either group (HSIT, pretraining 295,747 ± 202,422 and post-training 231,093 ± 64,853 cells (ml of whole blood)^−1^; MSIT, pretraining 295,101 ± 229,180 and post-training 223,693 ± 149,994 cells (ml of whole blood)^−1^), Fig 3c. Circulating CD34^+^ and CD309/KDR^+^ cells were increased in the HSIT group following training (pretraining 34 ± 37 and post-training 79 ± 125 cells (ml of whole blood)^−1^, Fig 3a), while they remained relatively unchanged with MSIT (pretraining 67 ± 80 and post-training 47 ± 47 cells (ml of whole blood)^−1^, Fig 3a); however, the interaction was not statistically significant (group × time interaction *P*= 0.32). Likewise, CD34^+^, CD309/KDR^+^ and CD133^+^ cells were increased in the HSIT group following training (pretraining 8 ± 6 and post-training 19 ± 23 cells (ml of whole blood)^−1^, Fig 3a), while they remained relatively unchanged after MSIT (pretraining 16 ± 18 and post-training 14 ± 12 cells (ml of whole blood)^−1^, Fig 3a); however, this also did not reach statistical significance (group × time interaction *P*= 0.33).

## Discussion

The major findings of the study were an increased L-FMC and total vessel reactivity following either MSIT or HSIT exercise training, which occurred without significant increases in FMD or CPC numbers. The altered L-FMC suggests a more sensitive vascular endothelium that exhibits an augmented response to acute reductions in blood flow (Gori *et al.* 2010) following interval training and that this adaptation was not dependent upon the metabolic stress induced by the training programme. Furthermore, a greater overall sensitivity to changes in blood flow of the vessel was observed (i.e. total vessel reactivity) following both training programmes. The findings also confirm the suggestion that increased FMD in the brachial artery is rarely observed with training of the lower limbs in relatively healthy participants.

Whether the metabolic stress is a determining factor in relation to changes in CPC numbers with exercise training is currently unclear (Sandri *et al.* 2005). Several research groups suggest that circulating progenitor cells are only mobilized from their various niches upon hypoxia-related signalling (e.g. by vascular endothelial growth factor; Sandri *et al.* 2005), while other researchers suggest these cells are mobilized either during exercise that is both below or above LT (Laufs *et al.* 2005; Cubbon *et al.* 2010). Few, if any, studies have attempted to isolate the effect of metabolic stress. It has been suggested that higher metabolic stress may cause greater production of reactive oxygen species, specifically H_2_O_2_, which would signal an upregulation of endothelial nitric oxide synthase protein (Lauer *et al.* 2005). As a result, CPC mobilization, which is nitric oxide dependent in humans (Cubbon *et al.* 2010), could be enhanced directly by higher metabolic stress. Yet clearly, this effect is rather short term and no longer apparent at 48 h postexercise.

This study benefited from careful matching of the amount of work completed throughout the 6 weeks of training. As the exercise intervals were completed at the same relative power output for both groups (intervals between 20 W and 120% of the peak work rate achieved in the ramp-incremental test), different interval durations were used to alter the metabolic stress (intensity) of the exercise sessions. This was described in detail by Turner *et al.* (2006), who showed that duty cycles lasting 30 s (10 s work and 20 s recovery) caused little accumulation of blood lactate. In contrast, a duty cycle lasting 90 s (30 s work and 60 s recovery) caused lactate accumulation and attainment of a raised, but stable lactate consistent with heavy-domain exercise (Rossiter, 2011). Using this model, our two groups were matched for training duration (30–40 min per session), relative power output and amount of work completed, but their overall metabolic stress was different. The finding that CPC numbers were similar in MSIT and HSIT in the present study, therefore, suggests that metabolic stress *per se* may not directly mediate their mobilization.

### Mechanisms of vascular adaptations

Low-flow-mediated constriction is a relatively new assessment of vascular function and relates to the endothelial contribution to resting vasomotor tone (Gori *et al.* 2010). As such, a greater comparative L-FMC response may represent greater sensitivity of the endothelium to reduced shear stress. Mechanistically, this may involve either the production of functional vasoconstrictors (eg. endothelin-1 or thomboxane) or a reduction in the production of vasodilators, including endothelium-derived hyperpolarizing factor, prostacyclin and other substances unrelated to nitric oxide (Spieker *et al.* 2003). Recent work (Hansen *et al.* 2011) would suggest that basal prostaglandin levels (thromboxane and prostacyclin) are reduced following 16 weeks of whole-body exercise training, which could alter the balance of vasoactive substances upon reduction of shear or the alteration of the shear profile (greater retrograde shear). This may result in enhanced coupling of artery diameter to shear following training. Similar to the increase in endothelium-derived hyperpolarizing factor with training, detraining through hindlimb unloading impairs vasoconstriction in conduit vessels, and this is endothelium dependent (Purdy *et al.* 1998). Thus, the initiation of regular exercise training may act in a reverse manner. Studies which determine the contribution of various mediators of the L-FMC response are warranted; however, the relationship between basal limb blood flow, which increased with training, and the L-FMC response suggests that the magnitude of the shear reduction upon cuff inflation may dictate the constriction, although the change in shear upon cuff inflation was not significantly different with training.

The increases in L-FMC were similar in MSIT and HSIT, which suggests that this training adaptation may occur through mechanisms that are independent of metabolic stress. This suggests that the total duration for which arterial shear stress is elevated, which was probably similar between the training groups, is a key determinant for vascular adaptations, and more so than metabolic factors. The training-induced increase in vasoconstriction could result from tighter coupling of the shear stimulus to endothelial vasodilator or constrictor substances, as outlined above. One other potential candidate could relate to circulating reactive oxygen species, which may induce vasoconstriction upon shear reduction. Elevated oxidative stress has been observed following high-intensity exercise training, contributing to reduced microvascular endothelial function (Bergholm *et al.* 1999). Further studies involving antioxidant administration or prostaglandin inhibition during the evaluation of L-FMC could elucidate the mechanism of these training-related changes.

The lack of an increase in brachial FMD with training is not uncommon, and a recent large cohort analysis of exercise training in young swine also failed to show any change in endothelium-dependent or -independent vascular function. This was further supported by the fact that there were no protein expression changes from arterial endothelial cell scrapes for enzymes involved in nitric oxide metabolism (Padilla *et al.* 2010). A lack of effect has been noted in several human studies (Green *et al.* 2004) and may be partly explained by the work of Tinken *et al.* (2008), who demonstrated that enhanced endothelial function occurs rapidly (within 2 weeks) upon initiation of an exercise training programme involving the forearm, but that this adaptation is supplanted by increases in vascular capacity (as determined by combined arterial occlusion and hand-grip exercise), which is evident at 8 weeks of training and returns endothelial function to pretraining levels (Tinken *et al.* 2008). In our study, the timing of post-training measures relative to any potential vascular capacity changes may contribute to our null findings. In addition, these measures were taken in the brachial artery and not the conduit vessels supplying the working muscle (unlike previous work that evaluated popliteal artery vascular function; Rakobowchuk *et al.* 2008), which is likely to contribute to the similarity in pre- and post-training FMD measures observed here. However, assessment of brachial artery vasomotor function as a surrogate for coronary artery vasomotor function remains important because it is non-invasive and may provide predictive value in cardiovascular disease risk assessments and alterations of vascular function with interventions (Celermajer *et al.* 1994). However, it should be noted that brachial vasomotor function does not represent systemic vasomotor function, which differs from limb to limb (Thijssen *et al.* 2011*b*).

In summary, vasomotor function increases with exercise training independent of the metabolic stress induced by the exercise session. In addition, these adaptations may have been masked in previous studies that simply evaluated the dilatory response to abrupt increases in shear and not vasoconstrictor responses to reduced shear.

### Circulating progenitor cells and exercise training in healthy participants

Our results indicate that interval training did not alter circulating progenitor cell numbers, as defined by CD34, CD133 and CD309/KDR antigen expression. Although there was a doubling of CD34^+^, CD309/KDR^+^ and CD34^+^, CD133^+^, CD309/KDR^+^ positive cells with HSIT training, and no change with MSIT, this trend was largely a result of two participants who displayed large increases (see Fig. 3). As measures were taken 48 h after the final exercise session, it is possible that lingering effects of the acute bout contributed to this variability, causing some participants to exhibit increases while most showed no change. The lingering acute effects of the last bout of exercise were originally suggested by Laufs *et al.* (2005), who tracked changes in CPC numbers up to 24 h of recovery following exercise above LT, and showed that CPC numbers could remain elevated for 24 h or more. In the present study, the HSIT group performed exercise involving a heavy-intensity metabolic stress, and in some participants a sustained rise in CPCs occurred following the final exercise session. As for the MSIT group, no discernable elevation was observed, suggesting that moderate-intensity exercise programmes are unlikely to induce a sustained elevation of CPC numbers. This suggestion is supported by Thijssen *et al.* (2006), who noted no elevations of CPCs with moderate-intensity training in older men. However, this may not be the case in patients with chronic disease, because several studies have shown elevated circulating CPCs beyond 48 h after training cessation (Adams *et al.* 2004; Sandri *et al.* 2005; Steiner *et al.* 2005). Taken together, the present data, and those of others (Adams *et al.* 2004; Sandri *et al.* 2005; Steiner *et al.* 2005), are consistent with the notion that lingering mobilization of CPCs may be a function of exercise intensity, suggesting a physiological drive to improve convective and diffusive oxygen delivery via enlargement of the capillary network in exercise above LT.

### Limitations

There are some limitations to the study that should be considered. Functional parameters of putative circulating progenitor cells were not assessed and may be more important than enumeration of these cells. The interpretation of L-FMC is difficult without invasive evaluations to determine mechanisms underlying the changes that occurred with training. With regard to the assessment of FMD, the Doppler insonation angle was not below 60 deg; however, it was identical at each testing session. This compromise was needed to ensure quality imaging, but may have increased the error in Doppler signal acquisition.

### Implications and clinical perspective

Alterations of vascular function with exercise training appear to go beyond simple changes of FMD and may also involve enhanced sensitivity to reductions in blood flow. It is possible that interval exercise training may be better tolerated in populations intimidated by the suggestion that continuous exercise is the preferred option, where alternatives may be needed. These populations may include those with various diseases and impairments, such as chronic obstructive pulmonary disease, chronic heart failure, peripheral vascular disease and those returning to health following acute coronary syndromes. Future clinical studies appear warranted.

## Conclusions

The present study has established that the metabolic stress of an interval exercise programme is not a determining factor for vascular endothelial adaptations. Neither heavy nor moderate metabolic stress improves FMD in apparently healthy young adults following a 6 week programme, although the basal endothelium reactivity is greater. This elevated endothelial reactivity manifests as a more pronounced vasoconstrictor response to decreased shear stress (L-FMC) and contributes to greater total vessel reactivity. The CPC numbers appear not to change with interval training, irrespective of metabolic stress of the exercise, although a transient prolonged elevation was noted in some participants who completed the heavy-intensity metabolic stress interval exercise training programme.
